# POLD3 as Controller of Replicative DNA Repair

**DOI:** 10.3390/ijms252212417

**Published:** 2024-11-19

**Authors:** Nabilah Alli, Anna Lou-Hing, Edward L. Bolt, Liu He

**Affiliations:** 1 School of Life Sciences, University of Nottingham, Nottingham NG7 2UH, UK; 2 Centre for Medicines Discovery, University of Oxford, Oxford OX3 7FZ, UK

**Keywords:** Polδ, Polζ, POLD3, histone, DNA replication, DNA repair, helicase

## Abstract

Multiple modes of DNA repair need DNA synthesis by DNA polymerase enzymes. The eukaryotic B-family DNA polymerase complexes delta (Polδ) and zeta (Polζ) help to repair DNA strand breaks when primed by homologous recombination or single-strand DNA annealing. DNA synthesis by Polδ and Polζ is mutagenic, but is needed for the survival of cells in the presence of DNA strand breaks. The POLD3 subunit of Polδ and Polζ is at the heart of DNA repair by recombination, by modulating polymerase functions and interacting with other DNA repair proteins. We provide the background to POLD3 discovery, investigate its structure, as well as function in cells. We highlight unexplored structural aspects of POLD3 and new biochemical data that will help to understand the pivotal role of POLD3 in DNA repair and mutagenesis in eukaryotes, and its impact on human health.

## 1. POLD3 in DNA Repair, Genome Stability, and Human Health

POLD3 is part of the eukaryotic B-family DNA polymerase complexes Polδ (polymerase delta) and Polζ (polymerase zeta) that synthesize DNA during stable chromosomal replication and during multiple modes of DNA repair [1,2,3]. In this review, we focus on POLD3 as a controller of DNA repair through physical and functional interactions within these polymerase complexes, post-translational modifications (PTMs), and interactions with other DNA replication–repair proteins. Most of these interactions and PTMs are within structurally flexible (intrinsically disordered) regions of POLD3 that are structurally unresolved, comprising 60–70% of the POLD3 protein [4,5,6,7,8,9,10].

**Figure 1:**
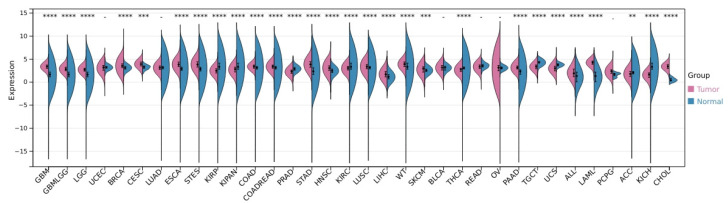
Pan-cancer analysis of POLD3 expression in tumor tissue (pink) compared with non-tumor tissue (blue). The levels of gene expression are upregulated in glioblastoma multiforme (GBM), glioblastoma and low-grade glioma (GBMLGG), brain lower grade glioma (LGG), breast invasive carcinoma (BRCA), cervical squamous cell carcinoma and endocervical adenocarcinoma (CESC), esophageal carcinoma (ESCA), stomach and esophageal carcinoma (STES), colon adenocarcinoma (COAD), colon adenocarcinoma/rectum adenocarcinoma esophageal carcinoma (COADREAD), stomach adenocarcinoma (STAD), head and neck squamous cell carcinoma (HNSC), lung squamous cell carcinoma (LUSC), liver hepatocellular carcinoma (LIHC), high-risk Wilms tumor (WT), skin cutaneous melanoma (SKCM), pancreatic adenocarcinoma (PAAD), acute lymphoblastic leukemia (ALL), acute myeloid leukemia (LAML) and cholangiocarcinoma (CHOL); and downregulated in kidney renal papillary cell carcinoma (KIRP), kidney renal clear cell carcinoma (KIRC), kidney chromophobe (KICH), pan-kidney cohort (KICH + KIRC + KIRP, KIPAN), prostate adenocarcinoma (PRAD), thyroid carcinoma (THCA), testicular germ cell tumors (TGCT), uterine carcinosarcoma (UCS), and adrenocortical carcinoma (ACC). ** *p* < 0.01. *** *p* < 0.001. **** *p* < 0.0001. Data were collected from the TCGA and GTEx, processed using Sangerbox3.0 [19].

There are several uncharacterized motifs of POLD3 that are important for maintaining genome stability and human health, mutations in those are associated with familial hearing impairment [11] and Omenn Syndrome [12].The significance of POLD3 in mammalian health is clear from the adverse effects on genome stability and fertility from deletions and knock-downs of POLD3 [13,14], and from Cancer Dependency mapping (DepMap [15]), and studies showing that it underpins hyperactive DNA synthesis in tumorigenesis [16,17] and spermatogenesis [18]. Our own analysis of human POLD3 data from the Cancer Genome Atlas (TCGA) and The Genotype-Tissue Expression (GTEx) project, showed alteration of POLD3 expression in 34 types of tumor (Figure 1).

POLD3 is necessary for DNA break repair, break-induced replication (BIR), and microhomology-mediated BIR (Section 4.2 and Section 4.3). These are processes that result in genetic instability, typified by tandem DNA duplications and point mutations, that are characteristic of cancer cells [20,21,22], and with mutagenic translesion synthesis more generally [23,24,25]. This review highlights what is known about how healthy cells control mutagenic BIR, and signposts new data and potential mechanisms.

## 2. Discovery of POLD3 in Yeasts and Mammals

**Figure 2:**
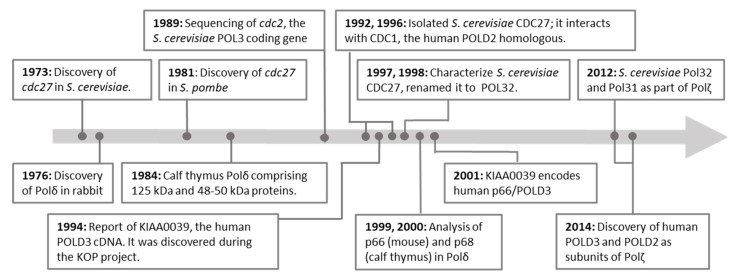
Timeline of POLD3 proteins studies. Nomenclature is described in the main text. We also refer to a timeline for DNA polymerases discoveries and studies provided by the Woodgate group: https://www.nichd.nih.gov/research/atNICHD/Investigators/woodgate/research/DNA-polymerases (accessed on 21 September 2024).

A timeline highlighting the discovery of POLD3 in several model systems is shown in Figure 2. POLD3 was discovered in *Saccharomyces cerevisiae*, named as *cdc27* in a strain giving faulty mitosis phenotypes [26]—over 100 *S. cerevisiae* ‘cell division cycle’ (cdc) strains that had been generated in a prior study using genome-wide mutagenesis from N-methyl-N′-nitro-N-nitrosoguanidine treatment [27]. Note that the product of the gene, CDC27, was renamed to Pol32 towards the end of 1990s. Strain *cdc27* was sensitive to low-temperature and had mating and cell division defects that were subsequently utilized in complementation tests by *Schizosaccharomyces pombe cdc* genes [28], identifying the *S. pombe cdc27* [29].

*S. pombe cdc27* transcripts gave an inferred CDC27 protein product [30] of 372-amino acids, but with no sequence homology with other proteins, reflecting that 60–70% of POLD3 is intrinsically disordered, detailed later. POLD3 proteins extracted from human, mouse, and chicken cells were called POLD3 or p66 (i.e., polypeptide of 66 kDa) [31,32], as p68 from Calf thymus [33,34], and as Pol32 and CDC27 proteins in *S. cerevisiae* and *S. pombe*, respectively [26,35,36,37]. CDC27 protein function was first reported by the Burgers group, using extensive biochemical fractionation of soluble proteins derived from protease-attenuated *S. cerevisiae* [38]. CDC27 was one of five purified protein subunits that fractionated with DNA synthesis and DNA exonuclease activity, referred to as yeast polymerase III in reference to its similarity with the biochemical activities of bacterial DNA polymerase III. Work in the 1990s established *S. cerevisiae* and *S. pombe* Polδ subunit composition, and the emergence of POLD3 in yeast, the POL32 (*S. cerevisiae*) and CDC27 (*S. pombe*), as a factor in mutagenic DNA repair—its deletion resulted in yeast cells hypersensitive to death from genotoxic agents, but with less mutagenesis of a reporter gene, revealing POLD3 as part of mutagenic DNA repair [37,39,40,41,42,43].

The mammalian Polδ complex had been isolated in 1976 from rabbit bone marrow cells as a high molecular mass DNA polymerase with 3′ to 5′ exonuclease activity [44]. It was named Polδ, following newly proposed nomenclature for all eukaryotic DNA polymerases [45]. Follow-up studies indicated Polδ comprised of 125 kDa and 48–50 kDa subunits—now called POLD1/p125 and POLD2/p50—with hints of additional subunits during fractionation [46,47]. Human “expressed sequence tag” databases available in the 1990s [48] gave the first sequence match to yeast POLD3 (CDC27) [49], although this was revealed later to be misalignment that had not identified bona fide POLD3. The mammalian POLD3 sequence was first identified from N-terminal amino acids of a ‘p66’ protein pulled-out from mouse cell extracts by immobilized PCNA (the replication ‘sliding clamp’ protein) [31]. N-terminal peptide sequencing matched p66 with the protein sequence deduced for *S. pombe* CDC27 [30], and with the predicted product of a human cDNA (KIAA0039) within the Kazusa ORFeome project (KOP) [50]. The monoclonal antibodies that were generated against relevant KOP cDNA product interacted with p66, supported the previous analysis [32]. Further sequence searching confirmed that calf thymus p68 and p66 were the same protein within mammalian Polδ [33,34].

POLD4 of Polδ was observed as a 22kDa protein from *S. pombe* during biochemical fractionation and then identified by sequencing as the *cdm1* gene product [35], a known suppressor of the *cdc1* allele that had been identified by mutagenesis [26]. Mammalian POLD4 was identified by the same route as POLD3; fractionation of soluble cellular proteins and protein sequencing that matched *S. pombe* Cdm1 [51]. A POLD4 subunit has not been identified in *S. cerevisiae*.

## 3. POLD3 Structure and Motifs

**Figure 3:**
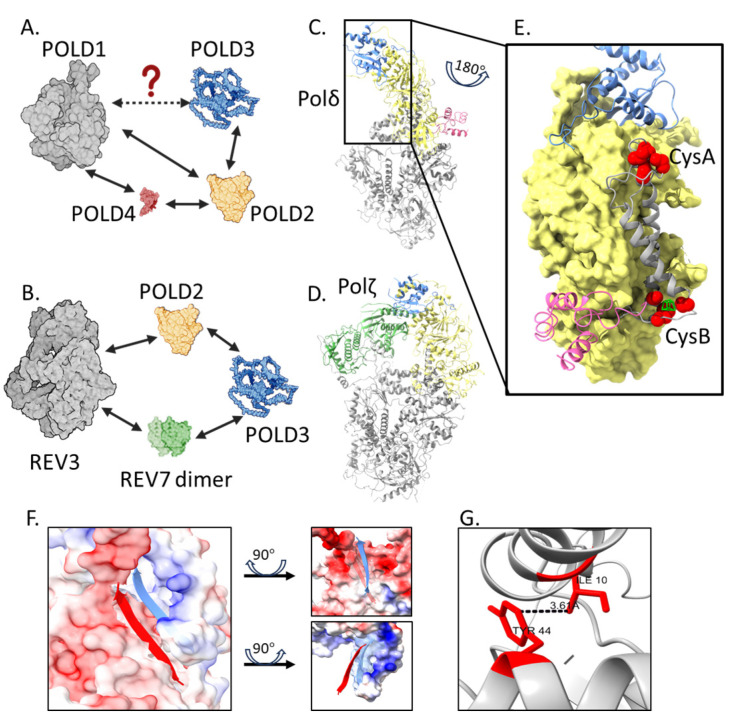
POLD3 in Polδ and Polζ complexes. (**A**,**B**) Experimentally determined interactions between subunits within Polδ and Polζ shown as solid arrows, although with a questionable interaction of POLD1 and POLD3. More detail about the experimental data can be found in Section 4 of the text; (**C**,**D**) Structures of human Polδ (PDB: 6TNY) and *S. cerevisiae* Polζ (PDB: 6V8P). The polymerase/nuclease catalytic subunits are in grey—POLD1 in Polδ and Rev3 in Polζ. POLD2 is yellow, and the partial structure of POLD3 (see detail in main text) is blue. POLD4 in Polδ is pink, and the Rev7 dimer of Polζ is green; (**E**) The completely resolved structure of human POLD2 (yellow) with its interaction to POLD3 (blue), POLD4 (pink) and two α-helixes of POLD1 (grey). The POLD2 CysA and CysB motifs are highlighted in red, and the iron–sulfur cluster of CysB (green) is thought to regulate POLD1 catalytic function; (**F**) Electrostatic surface of the POLD2–POLD3 interaction in human Polδ (PDB 6TNY), with negatively charged POLD2 and the β-sheet 447-CQPISFSG-454 in red, and positively charged POLD3 residues and β-sheet 73-SCHKVAVV-80 in blue; (**G**) POLD3 isoleucine-10 (ILE 10) highlighted in a clinical case report [11], may interact with POLD3 tyrosine-44 (TYR 44), a proposed substrate for phosphorylation. The minimum distance between the elements in two residues is 3.61Å. Images and evaluation of element distance were produced using ChimeraX from PDB 6TNY.

Structures and interactions of Polδ and Polζ complexes are summarized in Figure 3A–D. These revealed that POLD3 interacts with POLD2 of the polymerase catalytic core through a POLD3 winged helix–turn–helix (wHTH) fold (Figure 3E)—this fold is the only part of POLD3 that is structurally resolved, with the remaining 60–70% of POLD3 comprising intrinsically disordered protein regions (IDPRs) [3,4,5,6,7]. But, there is emerging knowledge about roles for the disordered regions. Here, we detail what is known with ideas for their functional significance.

### 3.1. Interaction of the POLD3 wHTH Fold

The interaction between POLD3 and POLD2 (Figure 3E,F) is essential for normal Polδ function—mutations of the POLD2-POLD3 interaction region in *S. cerevisiae* cause the *cdc27* cold sensitive phenotype [4]. The interaction centers on the POLD3 wHTH β3 (residues 73-SCHKVAVV-80) interacting with POLD2 β18 (447-CQPISFSG-454), stabilized by the positive–negative charged surface interaction of POLD3 and POLD2, respectively (Figure 3F) [4]. An isoleucine-10 to threonine mutation on the wHTH surface was associated with defects in hearing, immune development, and neurodevelopment in a patient described in 2023 [11]. Patients carrying the POLD3^I10T^ allele showed low POLD3 expression levels, as well as reduced POLD1 and POLD2 expressions in their peripheral blood mononuclear cells. This, together with evidence from other studies [14,52,53], indicated that the presence of POLD3 is necessary for protecting the integrity of the Polδ complex. The molecular basis for these multiple effects of POLD3^I10T^ is unknown. One line of enquiry notes that human POLD3 Ile-10 may form intramolecular contact with Tyr-44 (Figure 3G), a predicted target for regulatory phosphorylation [54] (see Section 5). We suggest perhaps the change of Ile10 to Thr mutation disrupts post-translational modification of POLD3, amplifying downstream effects in cells.

### 3.2. Conserved IDPR Regions of POLD3

**Figure 4:**
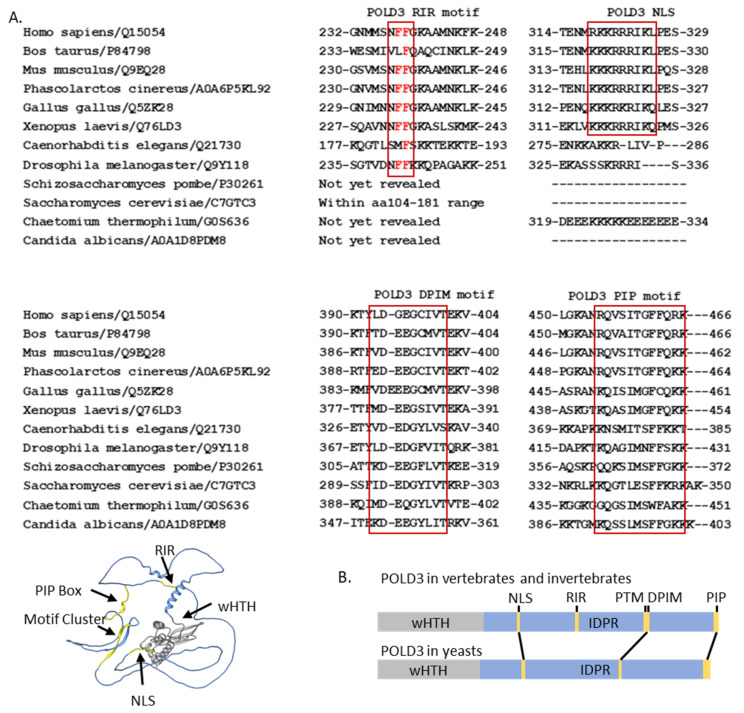
POLD3 has an N-terminus wHTH fold that is conserved across eukaryotes (see also Figure 3), and more C-terminal IDPRs containing some conserved motifs. (**A**) POLD3 alignment from 12 organisms framing in red squares the conserved amino acid motifs that have been studied: DNA polymerase interaction motif (DPIM); nuclear localization sequence (NLS), which seems to be present only in vertebrates and invertebrates but not in the fungi; Rev1 interaction region (RIR) typically formed by two hydrophobic phenylalanine amino acids; and PCNA interacting protein (PIP) motif. Those motifs (yellow) in POLD3 IDPR (blue) on a predicted POLD3 structure by Alphafold3, the wHTH fold of POLD3 is marked in grey. The organisms used were as follows: *Homo sapiens* (human), *Bos taurus* (cow), *Mus musculus* (mouse), *Phascolarctos cinereus* (koala), *Gallus gallus* (chicken,), *Xenopus laevis* (african clawed frog), *Caenorhabditis elegans* (roundworm), *Drosophila melanogaster* (fruit fly), *Schizosaccharomyces pombe* (fission yeast), *Saccharomyces cerevisiae* (budding yeast), *Chaetomium thermophilum* (thermophilic fungus) and *Candida albicans* (pathogenic yeast) for the sequence alignment. (**B**) Summary of the motifs of POLD3 proteins: The N-terminus resolved wHTH domain that is crucial for interaction with POLD2 (grey), and the unstructured C-terminus IDPR (blue), which contains the Rev1 interaction region (RIR), two nuclear localization signals (NLS), and a site containing a cluster of post-translational modifications (PTMs).

In the following content and also in Figure 4, we summarize the conserved regions from individual studies and give a brief introduction of their roles in protein–protein interactions and protein transport.

#### 3.2.1. The POLD3 Rev1 Interaction Region

The POLD3 Rev1 interaction region (RIR, 237-NFFGKAA-243) was identified through sequence similarity to consensus RIRs (‘NFFhhhh’, h denotes any alpha helix-forming residue) within the Y-family polymerases Polη, Polι and Polκ [55]. RIR recruits the Rev1 deoxycytidyl transferase protein that extends DNA over abasic DNA sites [56]. The mechanisms controlling the hand-off of Rev1 between its polymerase interactors is not clear, but surface plasmon resonance binding kinetics of Rev1 with RIR peptides, alongside structural analysis, has led to a model of ‘inserter’ and ‘extender’ subunit exchange [55]. Post-translational modifications may also control the dynamic interactions of RIR motifs and Rev1. ADP-ribosylation of Ser-236 and oxidative modification of Met-234 and Met-235 has been detected next to the human POLD3 RIR motif [57], but it has not been experimentally determined if these influence POLD3–Rev1 interaction. More detail about POLD3 post-translational modifications is provided in Section 6.

#### 3.2.2. The POLD3 DNA Polymerase Interacting Motif (DPIM)

The POLD3 DNA polymerase interacting motif (DPIM) within yeast POLD3 IDPRs interacts with Polα (Pol1) primase [58]. In *S. cerevisiae* POLD3, it is within residues 269–310 [59]. In *S. pombe*, mutations of the DPIM caused a loss of interaction with Polα–Pol1 and cell cycle defects [60]. The yeast POLD3 DPIM is conserved in mammalian POLD3 (residues 390–400, Figure 4A), though interaction with human Polα has not been shown. It has been proposed that the DPIM is embedded within an overall larger area of IDPR that is post-translationally modified, may control the interaction with Polα, detailed in Section 5.2.

#### 3.2.3. Two Nuclear Localization Signals

Human POLD3 may compare more than one nuclear localization signal in its IDPR. The identified NLS (human POLD3 319-KKRRRIKL-326) was highlighted in an NLS prediction program version 1.0 [61], then it was tested intracellularly and showed that mutations at this sequence abolished POLD3 transport into nucleus, with a reduction of 50% [62]. Re-analysis of human POLD3 sequences using a more recent NLS prediction (NucPred version 1.0 [63]), we identified a second putative NLS, 373-GENKRKRKR-385, which requires experimental investigation, located upstream of the DPIM, and conserved across all POLD3 homologues in vertebrates (Figure 4A).

A change of lysine-373 to threonine in the putative NLS was identified in a patient with Omenn’s syndrome [12,64]. The protein expressions of POLD1, POLD2, and POLD3 were unaltered in patient’s fibroblasts, indicating conserved physical integrity of the Polδ complex [12]. However, the fibroblasts showed cell cycle defects, most notably in progression to S-phase, consistent with a defect in initiating DNA synthesis during mitosis by an unknown mechanism.

#### 3.2.4. The C-Terminal PCNA Interaction Protein (PIP) ‘Box’ of POLD3 Proteins

The C-terminal PCNA interaction protein (PIP) ‘box’ of POLD3 proteins and also in POLD1 and POLD4—interacts with proliferating cell nuclear antigen (PCNA) protein, providing processivity of DNA synthesis by Polδ and Polζ. POLD3–PCNA interaction was first reported from *S. cerevisiae* [65]—similar PIP boxes had been characterized in FEN-1 and p21^Cip1^ [66], therefore the *S. cerevisiae* POL32 and *S. pombe* CDC27 versions could be identified and analyzed in vitro and vivo [36,37]. Interaction of human POLD3 with biotinylated PCNA was identified using purified proteins via pull-down and ELISA assays [67], and by observed colocalization of POLD3 and PCNA that was lost when POLD3 lacked the PIP box [62]. The POLD3–PIP box binding mechanism is conserved in structures of POLD3 peptides from human (e.g., in PDB: 1U76) and the thermophilic yeast *Chaetomium thermophilum* (PDB: 8P9O) [68,69]. Its key features are a POLD3 PIP-box glutamine within the PCNA Q-pocket, and isoleucine and phenylalanine residues within the PCNA hydrophobic pocket.

## 4. POLD3 as a Controller of DNA Synthesis by Polδ and Polζ

### 4.1. POLD3 Within Polδ and Polζ Complexes

The application of *Spodoptera frugiperda* cells (e.g., SF9) in producing milligram levels of human recombinant proteins started to emerge from the late 1990s, and this had been deployed to analyze the physical and functional interplay between each Polδ subunit. Complexes sufficiently stable to be isolated were: POLD1 + POLD4, POLD2 + POLD3, POLD1 + POLD2 + POLD4, and POLD1 + POLD2 + POLD3 [70,71]. These revealed regulatory roles of POLD3 on the polymerase and exonuclease activities within the Polδ core POLD1 + POLD2, coordinated with the other regulatory subunit, POLD4.

In vitro studies of these Polδ variations showed that POLD3 is significant in maintaining polymerase processivity and DNA lesion bypass. The absence of POLD3 triggers hyperactive DNA polymerization by POLD1–POLD2–POLD4 [70], but with reduced processivity [72]. POLD3 contributes to polymerase processivity through its binding with DNA [7], which may help to prevent Polδ from dissociating off DNA. POLD3 also facilitates the bypass of DNA lesions such as abasic sites by Polδ [23], especially if the proofreading function of POLD1 is also compromised [73], further indicating regulatory effects of POLD3 on DNA synthesis. In 2012, POLD2 and POLD3 were revealed to be part of Polζ in yeasts and humans [74,75,76]. POLD2-POLD3 with Rev7–Rev3 showed increased overall DNA synthesis across DNA cross-links [76] and increased DNA binding and processivity [77]. It was suggested that the positively charged unstructured region at the POLD3 C-terminus may contribute to binding DNA, though this has not been verified experimentally. POLD3 and Rev7 interact physically in *S. cerevisiae* Polζ [3,5] (Figure 3B), though it remains unknown if this is also the case in mammals. The *S. cerevisiae* Polζ holoenzyme structure is defined, e.g., [3], but human Polζ structures are currently limited to individual Polζ subunits and peptides [55,78,79].

It is not clear how POLD3 within the polymerase complexes exerts control on DNA synthesis. The lack of structural definition for most of POLD3 has limited insight into the extent of its other interactions, for example, if it physically interacts with POLD1 directly. *S. cerevisiae* POLD1 (aka POL3) and POLD3 (aka POL32) physically interact in yeast two-hybrid analysis [37], although POLD1–POLD3 complexes were not observed during size exclusion chromatography of *S. cerevisiae* proteins [80]. Though human POLD1 and POLD3 co-immunoprecipitated [67], whether they can form a stable complex independently from POLD2 and POLD4 is unknown. We speculate that modulation of POLD1 catalytic activity by POLD3 may be by influencing POLD2–POLD1 interactions, centered at CysA and CysB motifs of POLD1 (Figure 3E). These motifs are crucial for DNA synthesis by Polδ and maintain the integrity of an active complex [81,82]. Therefore, POLD3 may trigger disassociation of Polδ under certain circumstances, possibly through changes in POLD1–POLD2 interaction.

### 4.2. POLD3 in DNA Repair Synthesis by BIR

**Figure 5:**
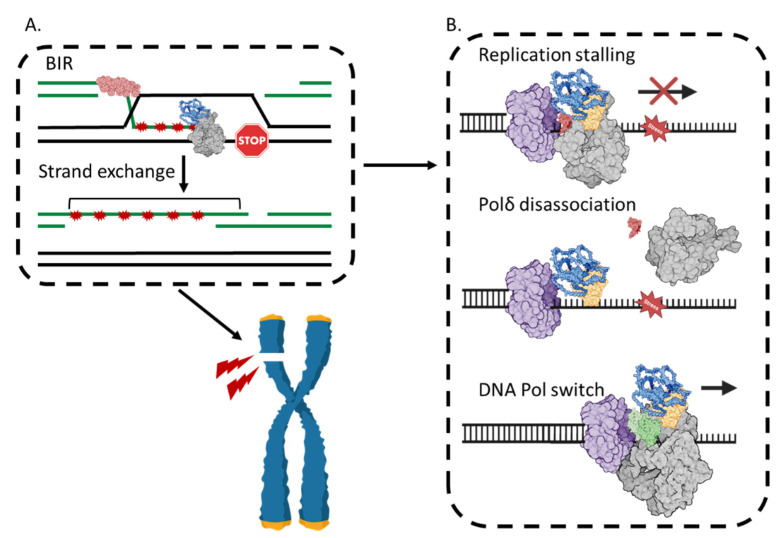
POLD3 is necessary for DNA repair by break-induced replication. (**A**) Shows DNA strand invasion catalyzed by a recombinase enzyme such as RAD51 (pink, bean-shape icon), which primes new DNA synthesis by Polδ (POLD3 colored blue, other subunits colored in grey). Mutations (red stars) are caused by DNA synthesis in this D-loop context, but the cell has survived a potentially lethal chromosome break; (**B**) A possible DNA polymerase switch from Polδ to Polζ. It is thought that during mutagenic DNA synthesis, in this context, the polymerase switches between Polδ and Polζ. This may come about by the PCNA (purple) interaction with POLD3 (blue)–POLD2 (yellow) core complex via the PIP motif at POLD3 C-terminus, with POLD1 (grey) and POLD4 (red) disassociating from the complex. The REV3L (grey)-REV7 heterodimer (green) forms a complex with POLD2–POLD3, thus Polζ proceeds DNA replication. This mechanism is likely one of the factors that causes mutagenesis during BIR.

BIR is DNA repair that uses DNA synthesis to complete repair of broken chromosomes [83,84,85,86], contributes to telomere maintenance and lengthening (alternative lengthening of telomeres, ALT) [87,88,89], and enables mitosis to complete chromosome duplication through difficult sequences (mitotic DNA synthesis, MiDAS) [90,91] (Figure 5).

BIR requires DNA Polδ [92,93,94], which synthesizes nascent DNA from a primer with a 3′OH end in a D-loop during recombination by ‘synapsis’ catalyzed by RAD51, or by DNA strand annealing by RAD52 or other emerging annealases; homologous recombination pathways were recently reviewed by other authors [95,96]. The outcome is new DNA that patches over the DNA break or gap. The critical role of POLD3 in BIR has been highlighted by depleting or deleting POLD3 in cells. Although human cells lacking POLD3 are sensitive to genotoxic agents—because BIR is absent—they show normal DNA repair by synthesis-dependent strand annealing (SDSA) or single-strand DNA annealing (SSA) [17], other major modes of replicative DNA repair. Further, loss of POLD3 suppresses genetic re-arrangement arising from BIR in *S. cerevisiae* and human cells—segmental duplications of chromosomes [17,20]. POLD3 also controls the onset of BIR within other repair pathways [97,98,99], including for repair of chromosomal ‘common fragile sites (CFS)’ [90,98] that also otherwise result in chromosomal re-arrangements.

### 4.3. BIR Can Be Mutagenic

Mutagenesis caused by POLD3-dependent BIR [17,20,22,100,101,102,103,104,105,106] belies the notion of ‘high fidelity’ DNA break repair by recombination. In human cells, BIR triggers point mutations clustered over 10s of kb (‘kataegis’) [107,108,109] and tandem duplications and re-arrangements of clusters of chromosomal regions (SDs and ‘chromothripsis’) [17,20,110]. The genetic changes are typified in ‘genomic disorder’, gene loss, and changes in gene copy number [111] characteristic of cancers. In this context, combined mutagenic BIR and MMBIR DNA synthesis are thought to predominate [17,110,112,113] if DNA repair using BRCA1/BRCA2 recombination pathways has become deregulated [114].

BIR differs from canonical eukaryotic replisomal DNA synthesis: a process Polδ synthesizes DNA lagging strands and a different polymerase, Polε, the leading strand [115,116]. Polδ in BIR is reviewed recently in reference [117]. Briefly, in BIR, Polδ synthesizes DNA from leading and lagging strand templates within a RAD51-generated ‘D-loop’, which becomes a ‘migrating bubble’ [94,118]. This de-couples leading and lagging strand synthesis as the ‘bubble’ advances with the aid of Pif1 helicase [119,120,121,122]. The tracts of ssDNA generated within the synthetic ‘bubble’ are available to re-prime ectopic DNA synthesis, are prone to DNA base damage, and provoke over-replication of inverted repeats (IRs, ‘fragile sites’) [123,124,125]. Point mutations and DNA duplications arise from BIR because of the Polδ DNA synthesis mechanism in this context, and by the switch to Polζ (MMBIR) from re-priming at short (<10 bp) DNA homologies.

A switch from BIR to MMBIR and, therefore, from Polδ to Polζ, is thought to occur when the advancing bubble is paused through loss of the Pif1 function [21], or in DNA repeat regions that re-prime DNA synthesis multiple times, generating the DNA tandem duplications characteristic of genomic disorders. The Polζ Rev3/Rev3L catalytic subunit was identified as causing point mutations during replicative DNA repair [126]. Its structure and function are reviewed by other authors in [127,128] and highlighted it as a major driver of mutagenic DNA repair in eukaryotes. For this, it requires POLD3 (with POLD2), which toggles between Polδ and Polζ complexes during lesion detection and extension of nascent DNA, revealed from biochemical and structural comparisons of the two complexes [3,6,7,8,74,75,76,129]. In Polδ, POLD3 modulates mutagenic DNA synthesis by POLD1 to become tolerant of base mismatches as a mutagenic translesion polymerase during DNA replication stress [23,73].

### 4.4. POLD3 as a Histone Chaperone During DNA Replication

**Figure 6:**
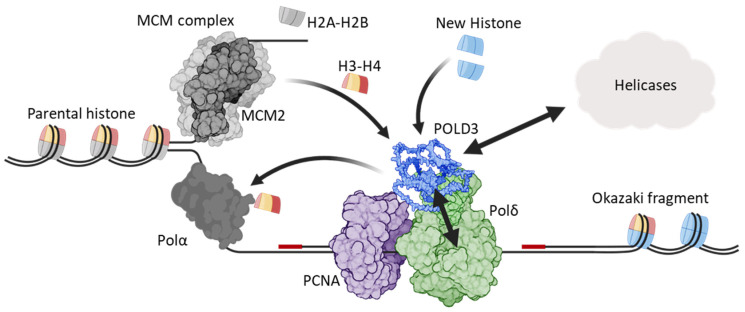
POLD3 exerts multiple functions during DNA replication. In this process, MCM2 of the MCM complex transfers parental histones H3-H4 to POLD3, which are then passed to Pol1 of Polα. In addition, POLD3 can independently interact with newly synthesized H3-H4. In parallel with histone disposal, POLD3 also regulates the catalytic function of Polδ/POLD1 and interacts/coordinates with DNA repair helicases—see main text such—highlighted as double arrows.

DNA replication at replisomes requires coordinated transfer of histone nucleosomes between parental and nascent DNA leading and lagging strands (reviewed in [130])—a process very recently implicating POLD3 in *S. cerevisiae* [131,132,133]. In this context, POLD3 (POL32) acts as a hub of interaction to enable the histone dimer H3-H4 of the parental DNA nucleosome to transfer to DNA lagging strands (Figure 6). POLD3 physically binds to H3-H4, receiving it from MCM2 helicase, and passing H3-H4 to the Pol1 subunit of the lagging strand polymerase, Polα [131]. The molecular mechanism of the POLD3/H3-H4 interaction is not known, but combining data from two studies [131,132] suggests that in *S. cerevisiae* POLD3 amino acid regions 244–270 and/or 309–350 are required for histone binding. Mutating the PCNA interacting motif (PIP box) of POL3 (POLD1) also compromised the histone transfer [133], probably by disrupting Polδ processivity with disruption of the PCNA–POL3/POLD1 interaction. The discovery of this novel POLD3 function broadens its critical roles as a regulator of eukaryotic genome stability, from DNA repair synthesis of Polδ and Polζ to chromatin organization during stable DNA replication.

## 5. Post-Translational Modifications of POLD3

Post-translational modifications (PTMs) of POLD3 are catalogued in the *Saccharomyces* Genome Database (SGD, https://www.yeastgenome.org), which cites studies from 2008 to 2021, identifying 18 phosphorylated or SUMOylated amino acids in *S. cerevisiae* POLD3. Human POLD3 has been detected with phosphorylation, ubiquitination, SUMOylation, serine ADP-ribosylation, and oxidative modifications. We round-up the data here, and add data gleaned from proteome-wide mass spectrometry repositories across several human cell types, but that currently have no associated functional or structural data—we highlight examples for further investigation.

### 5.1. SUMOylation of POLD3

Human POLD3 was SUMOylated when co-expressed with SUMO3 in U2OS cells [134]. In this study, mutation of two residues (POLD3 Lys-258 and Lys-433) with highest scores for predicted SUMOylation in SUMOplot™ (https://www.abcepta.com/sumoplot, accessed on 21 September 2024) resulted in loss of SUMOylation. Lys-258 and Lys-433 POLD3 residues were also SUMOylated in a screening method in human cells that returned 14,869 SUMOylation sites proteome-wide [135]. This study also provided an additional nine SUMOylated amino acids in POLD3, including two in the POLD3 wHTH domain [135]. In both studies, using in vitro biochemistry [134] and proteomics [135], POLD3 was SUMOylated only by SUMO3. Another study [136] saw no SUMOylation of POLD3 when co-expressed with only SUMO1 or SUMO2. However, POLD3 was a target for SUMO2 modification in cells undergoing replication stress through treatment with the B-family polymerase inhibitor aphidicolin [137] and when extracted in iPOND studies of proteins associated with nascent DNA synthesis [138].

### 5.2. Phosphorylation

POLD3 was phosphorylated within the Polδ complex extracted from mouse cells and human cells [67,139]. Phosphorylation of isolated human POLD3 maps to the amino acid region 384 to 399 (Figure 4B), which contains the potential targets for phosphorylation Ser-389 and Thr-391, where Thr-391 is part of a casein kinase II (CK2) phosphorylation targeting motif (XS/TXXD/E) [139,140]. A POLD3 peptide spanning this region could indeed be phosphorylated in vitro by CK2 at this threonine residue [139]. The location of this phosphorylated POLD3 region within its DPIM motif suggests that it may regulate interaction with Polα-Pol1. Phosphorylation of POLD3 modulates interaction with PCNA at POLD3 PIP-box residues Ser-458 and Thr-460—phosphorylation destabilized the interaction in pull-down assays and decreased processive DNA synthesis by Polδ-PCNA holo-complex, providing potential control of DNA replication in cells [139,141].

A proteome-wide mass spectrometry analysis of HeLa cell extracts after stimulation using epidermal growth factor (EGF) identified 2244 proteins with 6600 phosphorylated sites, including POLD3 (142). POLD3 was phosphorylated at serine residues Ser-307, Ser-407, Ser-409 and Ser-413, and Thr-311 and Thr-411, which are predicted to be phosphorylated by casein kinase II (CK2) [142]. This mapping discrepancy with the in vitro study may arise from different surface exposed serine and threonine residues in isolated POLD3 (in vitro) compared with POLD3 in polymerase complexes in cells. And, phosphorylated residues of POLD3 in response to EGF may differ from EGF-independent DNA processing. Indeed, POLD3 Ser-458, which was phosphorylated in vitro [139], was ADP-ribosylated when cells were stressed using hydrogen peroxide [57].

### 5.3. ADP-Ribosylation

Multiple ADP-ribosylation sites have been detected in human POLD3 [57,143,144], and there seem to be at least two different ADP-ribosylation events targeting POLD3, in response to whether or not cells are experiencing DNA damage [145]. Mutation of Ser-422-Ala in human POLD3 had no effect on ADP-ribosylation before the onset of DNA damage, but resulted in no ADP-ribosylation of POLD3 after DNA damage. This Ser-422 to-Ala change corresponded with a failure of DNA repair by BIR in response to hydroxyurea treatment, which is hypothesized to be caused by the mutant POLD3 failing to organize at chromatin. It will be informative to study the functional consequences of these and other PTM sites in POLD3 on Polδ catalytic activities, and its interactions with DNA repair proteins other than PCNA.

## 6. Interactions of Polδ and POLD3 with DNA Repair Helicases

### 6.1. Srs2, BLM, WRN and HELQ

POLD3 interacts physically and functionally with the DNA repair helicases such as SF1 family helicase Srs2, the RecQ-family helicases WRN and BLM, and the Ski2-family DNA helicase-annealase HELQ [146,147,148,149,150,151]. The POLD3–Srs2 interaction was detected in *S. cerevisiae* at Srs2 aa 1132–1175, using Srs2 fragments in two-hybrid reporter assays [146,152], although the interaction was not detected using full-length Srs2 [59]. Purified *S. cerevisiae* WRN protein stimulates primer extension by *S. cerevisiae* Polδ, but had no effect on other polymerases; an effect that required Pol32 (POLD3) and may be controlled by interaction of WRN with POLD2 [151]—Polζ, which also requires POLD3, was not tested [147]. Stimulation by WRN of Polδ DNA synthesis acted at initiation of primer extension, probably reflecting WRN removing secondary DNA structures (the study used tetraplexes) that otherwise block Polδ [148]. In human cells, BLM helicase promotes BIR-like/POLD3-dependent DNA synthesis when R-loops accumulate, and at telomeres [149,153,154,155], suggesting those two proteins may physically interact in cells.

In vitro assays showed interaction of Polδ and POLD3 with HELQ, which halted DNA synthesis, an effect not caused HELQ physically binding to DNA—HELQ mutants deficient at DNA binding were fully proficient at inhibiting Polδ [150]. Physical interaction was mapped to a 200 amino acid HELQ IDPR [150] and required an IDPR region of POLD3 that is rich in basic residues (authors’ unpublished data). Inhibition of Polδ DNA synthesis by HELQ was accompanied by stimulation of the HELQ single-strand DNA annealing activity, which was further stimulated when POLD3 was used alone instead of the Polδ complex. The significance of these in vitro observations to DNA repair in cells is not known, but alongside known physical and functional interaction of HELQ with RPA and Rad51 [120,156,157], and the genetic re-arrangements arising from loss of HELQ in cells [158], HELQ is emerging as a controller of the genetic outcomes of DNA repair and recombination in metazoans (HELQ is absent in yeasts).

### 6.2. Novel POLD3–Helicase Interactions Revealed During Cas9 Nuclease Screening

RNA-guided genome editing using bacterial Cas9 nuclease relies on host cell DNA repair to fulfil the editing by recombination and new DNA synthesis. The unpredictable genetic outcomes of Cas9-induced DNA repair have driven attempts to control it, to favor editing outcomes that are high fidelity. This has included using Cas9 proteins fused to proteins that rapidly recruit DNA repair enzymes to Cas9 DNA cut sites. One such study [159] identified that a Cas9–POLD3 fusion was especially effective at stimulating recombination-dependent DNA repair in response to Cas9 DNA cutting. They fused Cas9 with POLD3 lacking wHTH residues 1–106, which interact with POLD2. Using this construct and proximity-dependent biotin labelling, reviewed in [160], they identified Cas9 and Cas9–POLD3 interactors after Cas9-mediated DNA cutting, producing 76 novel interactors of POLD3–Cas9 that were absent from Cas9 alone—these potential POLD3 interactions during DNA break repair can be viewed in the supplementary data of that study. Another recent study [161] used multiplex co-fractionation/mass spectrometry (CF/MS), evaluating protein–protein interactions of human POLD3 that included PCNA and POLA1 (the catalytical subunit of human Polα), as expected, but also suggested novel physical associations with several DNA replication and repair proteins, listed in the supplementary data of that study.

## 7. Concluding Comments

POLD3 is essential for eukaryotic stable DNA replication and replicative DNA repair. In the latter, its ability to synthesize DNA at sites of DNA breaks underpins cell survival, but at the cost of mutagenesis. The multiple interactions of POLD3 within Polδ and Polζ polymerase complexes and with other DNA processing proteins, and its predominantly disordered structure, suggest that it is a flexible DNA repair ‘hub’ for controlling replicative repair of broken DNA.

## Data Availability

Data are contained within the article.
